# Complete mitochondrial genome of *Cacospongia mycofijiensis* (Dictyoceratida: Demospongiae): the first report for the sponge family Thorectidae

**DOI:** 10.1080/23802359.2016.1186513

**Published:** 2016-07-10

**Authors:** Hiroaki Aoyama, Seikoh Saitoh, Sanghwa Park, Yumi Shirai, Naoya Shinzato

**Affiliations:** a Center of Molecular Biosciences, Tropical Biosphere Research Center, University of the Ryukyus, Nishihara, Okinawa, Japan;; b Technology Research Association for Next Generation Natural Products Chemistry, Koto-Ku, Tokyo, Japan

**Keywords:** *Cacospongia mycofijiensis*, complete mitochondrial genome, Demospongiae, illumina sequencing, phylogeny

## Abstract

We sequenced a complete mitochondrial genome of the marine sponge, *Cacospongia mycofijiensis*, which is the first report for the family Thorectidae in the class Demospongiae. The mitogenome was obtained from a *de novo* assembly of shotgun genome sequencing using Illumina Miseq technology, which reconstructed a circular genome with 97 × of sequence coverage. The assembled mitochondrial genome consisting of 16,227 bp includes 14 protein-coding genes, 2 rRNAs and 2 tRNAs. This complete mitogenome sequence will be useful especially for the phylogenic studies of Demospongiae.

The class Demospongiae (Metazoa, Porifera) is the largest and the most diverse class of the phylum Porifera, which represents one of the most challenging groups in animal phylogenetic relationships still being unresolved (Boury-Esnault 2006; Van Soest et al. 2012). Recently, the complete mitochondrial genome data were proposed as an effective tool for molecular phylogeny studies of Demospongiae (Lavrov et al. 2008). The genus *Cacospongia* (Thorectidae, Dictyoceratida) contains 36 species and some species were reported to produce bioactive secondary compounds (Rubio et al. 2007; Van Soest et al. 2016). Particularly, the species *Cacospongia mycofijiensis* (Kakou et al. 1987) is known to generate multiple bioactive polyketide compounds in the southwest pacific ocean (Quinoa et al. 1988; Johnson et al. 2007). This mitochondrial genome should be useful for phylogenic studies in Demospongiae; moreover, it may contribute to chemical product researches in *Cacospongia*.

The *C. mycofijiensis* specimen was collected from the sea cave near Iejima Island (Ohoba No.2 cave, 26.7244 N 127.8297 E) at 30 m depth in June 2014, which was sequenced by whole-genome shotgun sequencing with illumina MiSeq (Illumina Inc. San Diego, CA) via paired-end library (75bp ×2). The mitogenome was *de nove* assembled using the Newbler v 2.7 software (Roche Diagnostics, Basel, Switzerland). The contigs of mitogenome were identified by BLAST search against GenBank database (Benson et al. 2015). Gene annotations were predicted using MITOS (Bernt et al. 2013) and were manually refined by using ORFfinder (http://www.ncbi.nlm.nih.gov/projects/gorf/). As additional information for vouchering, we sequenced two ribosomal RNA genes (18S and 28S) of the identical specimen by Sanger sequencing (GenBank accession no. LC136933 and LC136934). The leftover specimen is deposited in the Ryukyu University Museum, Fujukan (RUMF) (accession no. RUMF-ZP-00016).

The resulting mitogenome (GenBank accession no. LC133169) is 16,227 bp long with 97 × of the sufficient read coverage. The G + C content of the mitogenome is 37.1%. We identified 2 rRNAs, 2 tRNAs (*trnW* and *trnM*), and 14 protein-coding genes including the subunit 9 of ATPase synthase, which is a common gene in Demospongiae (Lavrov et al. 2008). The overall gene organization is consistent with the conserved order among Dictyoceratida sponges.

We performed a phylogenetic analysis using 13 complete mitochondrial genomes of Demospongiae. We concatenated the 14 protein-coding genes and 2 rRNA genes, and employed the maximum-likehood approach with MEGA7 (Kumar et al. 2016) using the GTR + GAMMA model for the tree construction. The node support was calculated with 1000 bootstrap replicates. As the result, *C. mycofijiensis* was placed in a cluster of the order Dictyoceratida and in the clade of the subclass Keratosa (Figure 1). It confirms the phylogenetic placement of the order Dictyoceratida evaluated by Lavrov et al. (2008). Moreover, this tree suggests that the family Thorectidae forms a distinct clade from the other families, Verticillitidae, Irciniidae and Spongiidae, which were not distinguished by CO1 and 28S rRNA gene trees (Erpenbeck et al. 2012).

**Figure 1.**
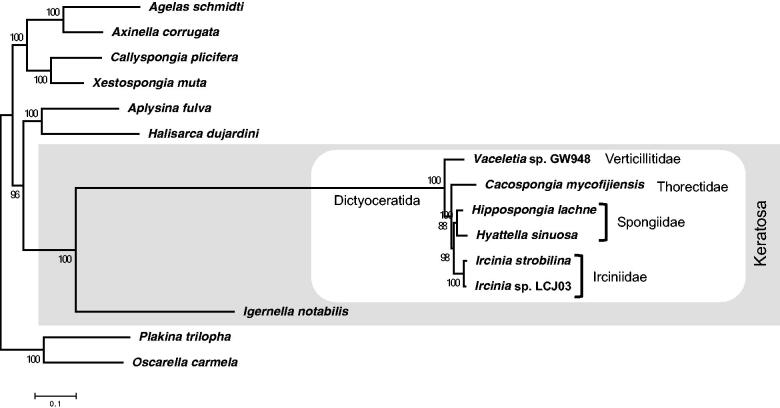
Phylogenic analysis of the order Dictyoceratida in the class Demospongiae using concatenated gene sets in mitochondrial genome. A maximum likelihood tree is based on 16 mitochondrial loci (cob, cox1, cox2, cox3, nd1, nd2, nd3 nd4, nd4l, nd5, nd6, atp6, atp8, atp9, rrnL and rrnS) of the 13 species, which were completed mitochondrial genome, including *Cacospongia mycofijiensis* (LC133169), *Vaceletia* sp. GW948 (NC_010218.1), *Ircinia strobilina* (NC_013662.1), *Ircinia* sp. LCJ03 (KC510274.1), *Hippospongia lachne* (NC_010215.1), *Hyattella sinuosa* (NC_021422.1), *Igernella notabilis* (NC_010216.1), *Halisarca dujardini* (NC_010212.1), *Aplysina fulva* (NC_010203.1), *Callyspongia plicifera* (NC_010206.1), *Xestospongia muta* (NC_010211.1), *Agelas schmidti* (NC_010213.1), and *Axinella corrugata* (NC_006894.1), with *Oscarella carmela* (NC_009090.1) and *Plakina trilopha* (NC_014852.1) as the outgroup. The tree is based on the General Time Reversible + Gamma site (GTR + G) model of the nucleotide substitution. The numbers at the nodes are bootstrap percent probability values based on 1,000 replications. White box: the clade of the order Dictyoceratida; Gray region: the clade of the subclass Keratosa.

## References

[CIT0001] Benson DA, Clark K, Karsch-Mizrachi I, Lipman DJ, Ostell J, Sayers EW 2015 GenBank. Nucleic Acids Res. 43:D30–D35. 25414350 10.1093/nar/gku1216 PMC4383990

[CIT0002] Bernt M, Donath A, Jühling F, Externbrink F, Florentz C, Fritzsch G, Pütz J, Middendorf M, Stadler PF 2013 MITOS: improved de novo metazoan mitochondrial genome annotation. Mol Phylogenet Evol. 69:313–319. 22982435 10.1016/j.ympev.2012.08.023

[CIT0003] Boury-Esnault N 2006 Systematics and evolution of demospongiae. Can J Zool. 84:205–224.

[CIT0004] Erpenbeck D, Sutcliffe P, Cook Sde C, Dietzel A, Maldonado M, van Soest RW, Hooper JN, Wörheide G 2012 Horny sponges and their affairs: on the phylogenetic relationships of keratose sponges. Mol Phylogenet Evol. 63:809–816. 22406528 10.1016/j.ympev.2012.02.024

[CIT0005] Johnson TA, Tenney K, Cichewicz RH, Morinaka BI, White KN, Amagata T, Subramanian B, Media J, Mooberry SL, Valeriote FA, Crews P 2007 Sponge-derived fijianolide polyketide class: further evaluation of their structural and cytotoxicity properties. J Med Chem. 50:3795–3803. 17622130 10.1021/jm070410z PMC2772109

[CIT0015] Kakou Y, Crews P, Bakus GJ 1987 Dendrolasin and latrunculin A from the Fijian sponge *Spongia mycofijiensis* and an associated nudibranch *Chromodoris lochi*. J Nat Prod. 50:482–484.

[CIT0006] Kumar S, Stecher G, Tamura K 2016 MEGA 7: molecular evolutionary genetics analysis version 7.0 for bigger datasets. Mol Biol Evol. 28:2731–2739. 10.1093/molbev/msw054 PMC8210823 27004904

[CIT0007] Lavrov DV, Wang X, Kelly M 2008 Reconstructing ordinal relationships in the demospongiae using mitochondrial genomic data. Mol Phylogenet Evol. 49:111–124. 18583159 10.1016/j.ympev.2008.05.014

[CIT0008] Quinoa E, Kakou Y, Crews P 1988 Fijianolides, polyketide heterocycles from a marine sponge. J Org Chem. 53:3642–3644.

[CIT0009] Rubio BK, van Soest RW, Crews P 2007 Extending the record of meroditerpenes from *Cacospongia* marine sponges. J Nat Prod. 70:628–631. 17346077 10.1021/np060633c

[CIT0010] Van Soest RW, Boury-Esnault N, Hooper JNA, Rützler K, de Voogd NJ, Alvarez de Glasby B, Hajdu E, Pisera AB, Manconi R, Schoenberg C, et al World Porifera database. [Internet]. 2016 [cited 2016 March 8]. Available from: http://www.marinespecies.org/porifera.

[CIT0011] Van Soest RW, Boury-Esnault N, Vacelet J, Dohrmann M, Erpenbeck D, De Voogd NJ, Santodomingo N, Vanhoorne B, Kelly M, Hooper JN 2012 Global diversity of sponges (Porifera). PLoS One. 7:e35105. 22558119 10.1371/journal.pone.0035105 PMC3338747

